# Spider bites of medical significance in the Mediterranean area: misdiagnosis, clinical features and management

**DOI:** 10.1590/1678-9199-JVATITD-2019-0100

**Published:** 2020-10-02

**Authors:** Gabriele Fusto, Luigi Bennardo, Ester Del Duca, Daniela Mazzuca, Federica Tamburi, Cataldo Patruno, Steven Paul Nisticò

**Affiliations:** 1 Unit of Dermatology, Department of Health Sciences, Magna Graecia University, Catanzaro, Italy.; 2 Department of Dermatology, Mount Sinai Medical Center, New York, USA.; 3 Unit of Forensic Pathology, Department of Health Sciences, Magna Graecia University, Catanzaro, Italy.

**Keywords:** Spider bites, Diagnosis, Diagnostic errors, Venoms

## Abstract

Despite the disrepute spiders have had for centuries, their bite is a rare occurrence. In the Mediterranean area, only two of the numerous known species are considered of medical significance: *Latrodectus tredecimguttatus* and *Loxosceles rufescens*. Spider bites have no pathognomonic signs or symptoms, therefore most diagnoses are presumptive; a spider bite can only be diagnosed when a spider (seen at the time of the bite) is collected and identified by an expert, since most physicians and patients are unable to recognize a certain spider species or distinguish spiders from other arthropods. Skin lesions of uncertain etiology are too often attributed to spider bites. In most cases, these are actually skin and soft-tissue infections, allergic reactions, dermatoses etc. Misdiagnosing a wound as a spider bite can lead to delays in appropriate care, cause adverse or even fatal outcomes and have medical-legal implications. Concerningly, misinformation on spider bites also affects the medical literature and it appears there is lack of awareness on current therapeutic indications for verified bites.

## Background

Spiders are eight-legged arthropods belonging to the class of arachnids (Arachnida); they can be found worldwide in nearly every habitat. Most spiders, when under threat, flee or pretend to be dead (a behavior known as thanatosis). Because of their nature, they rarely bite humans and only do so when they cannot escape or find themselves pressed against the skin (to avoid being pierced by their fangs, unwanted spiders should be flicked away rather than squashed). Unfortunately, there is a lack of epidemiological studies on this matter in European countries. The rarity of spider bites is evident when considering, for instance, that their annual frequency in Switzerland is estimated on average at 55 per million inhabitants, and only 2% of them are brought to medical attention [1]. Moreover, a study from Turkey reports that only 82 cases were referred to their National Poison Information Center from 1995 to 2004, most of which occurred during summer [2].

Unlike hematophagous insects, spiders have no interest in approaching humans, since they feed on other invertebrates. Bill Shear, biology professor and former president of the American Arachnological Society, in an interview to Scientific American said “Spiders regard us much like they’d regard a big rock, we’re so large that we’re really just part of the landscape” [3]. Differently from other arthropods, spiders do not usually transmit communicable diseases to humans and play an important role in our ecosystem by regulating the population of insects, therefore they should be considered allies rather than foes.

As of 2020, there are over 48000 recognized species of spiders [4], of which more than 5000 have been observed in the Mediterranean area [5] (1671 in Italy alone) [6]. Despite this rich biodiversity and the disrepute these animals have had for centuries, only the bite of few species is considered of medical significance, while others usually cause mild symptoms (comparable to those resulting from a mosquito sting).

The severity of the clinical picture depends on the type and quantity of injected venom and potential infections. It is known that females can store a greater volume of venom in their glands [7], whereas some spiders can bite without injecting any venom at all, inflicting the so-called “dry bites”. Bacteria such as *Clostridium perfrigens* [8, 9] and methicillin-resistant *S. aureus* (MRSA) [10] have been identified in many cases of *Loxosceles* bites and are considered accountable for the most severe dermonecrotic lesions. Initially, spiders were thought to be vectors of these bacteria. However, in a 2006 study [11], over 100 common house spiders were collected and none was found to carry *Staphylococcus aureus* or MRSA on their fangs. Therefore, MRSA colonization may be secondary to the spider bite, if not completely unrelated.

Regardless of the type of venom, in predisposed individuals, a spider bite may trigger major allergic reactions. These reactions include generalized pruritus and erythema, lymphangitis, immune-mediated hemolytic anemia, and acute generalized exanthematous pustulosis (AGEP) [12, 13, 14, 15]. In patients with a chronic autoimmune disease, a spider bite may trigger acute exacerbations (as seen in a woman with systemic lupus erythematosus) [16]. A case of *Herpes zoster* developing after a spider bite and resulting in residual paresthesia has also been reported [17].

Death by a spider bite is exceptionally rare and only two cases involving a European species can be found in the literature [18, 19]. The Australian funnel-web spiders (family Atracidae, of which the most famous species is *Atrax robustus*) are thought to be the most dangerous spiders to humans, but despite 198 bite reports (of which 77 with a severe envenomation), only 13 deaths were recorded, and all occurred before the introduction of an antivenom in 1981 [20]. Along with *Atrax robustus*, another spider considered highly venomous is *Phoneutria nigriventer* (known as the Brazilian wandering spider). Since 1903, 15 deaths following its bite have been reported in Brazil, but in only two cases there were sufficient details to confirm a causal nexus [21].

Of the few species considered of medical significance, whose bite may have severe complications, only two can be found in the Mediterranean area: *Latrodectus tredecimguttatus* (known as the Mediterranean black widow) and *Loxosceles rufescens* (known as the violin spider), which is native to Europe but has been spread worldwide.

The aim of the present article is to represent the current state of the art for spider bite management in the Mediterranean area and provide healthcare professionals with the fundamental knowledge needed to confront a suspected case.

## Materials and Methods

**Figure 1.**
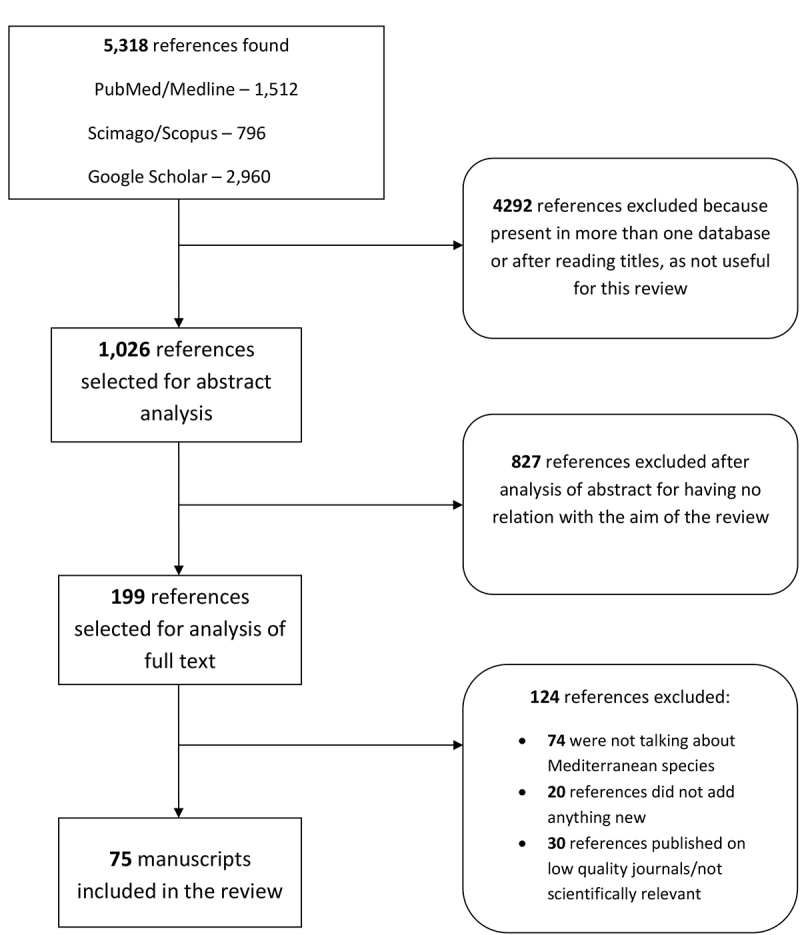
Article selection flow chart.

Different search engines were used for this article: PubMed, Google Scholar and Scimago-Scopus. Keywords searched include “spider bite”, “*Loxosceles*”, “*rufescens*”, “*Latrodectus*”, “*tredecimguttatus*” AND “therapy”, “treatment”, “diagnosis”. Articles concerning non-European mygalomorphs (commonly kept as pets by enthusiasts) were excluded from the review and those concerning American and Australian spiders were limited to the most relevant cases, involving related species (*Loxosceles* spp. and *Latrodectus* spp.) or species found in Europe as well (such as *Steatoda grossa*). Articles that did not bring any new information were excluded after a full reading. The article selection flow chart is better described in Figure 1. Other information was obtained through online resources dedicated to spiders (such as “World Spider Catalog”, “Aracnofilia” and “araneae - Spiders of Europe”). 

## Misdiagnosis

The habit of attributing skin lesions of uncertain etiology to spider bites is not limited to Italy or the modern age and is popular among healthcare professionals too, probably because of the countless myths surrounding these animals and their classic association with horror in movies and novels, which also contributed to making arachnophobia widespread. Spider bites have no pathognomonic signs or symptoms; therefore, most diagnoses are presumptive. A spider bite can only be diagnosed when a spider (seen at the time of the bite) is collected and identified by an expert, since most physicians and patients are unable to recognize a certain spider species or distinguish spiders from other arthropods.

Most of the alleged spider bites turn out to be skin and soft-tissue infections, allergic reactions, dermatoses, signs of neoplasia and wounds caused by physical or chemical agents or by other arthropods (usually blood-sucking bugs such as mosquitoes and ticks - accountable for several conditions, including Lyme disease). Lyme disease can cause local and systemic symptoms that are similar to those resulting from a *Loxosceles* bite and therefore is sometimes misdiagnosed as such [22]. Such disease should always be considered in endemic areas when performing a differential diagnosis for a suspected spider bite. Tularemia may also mimic necrotic arachnidism [23]. Lesions showing signs of necrosis are most likely to be attributed to a spider bite, probably because it is known that the venom of some spiders can cause dermonecrotic lesions. In the USA, dermonecrotic wounds of uncertain etiology are often attributed to the brown recluse spider (*Loxosceles reclusa*, a relative of the Mediterranean *L. rufescens*) and many such diagnoses occur in parts of the country where the spider is not native [24].

Pezzi et al. [25] reported a case of necrotic arachnidism in a woman “with no history of diabetes and allergies” and a mild form of myasthenia, stating that the patient had been bitten by a spider while cleaning a cellar the evening before hospitalization. The spider was not captured and its identification as *L. rufescens* was merely based on “the description and place where the bite occurred”. Additional investigations done by Nentwig, Pantini and Vetter [26] at the National Poison Control Centre in Milan revealed that the patient was actually diabetic and suffered from an infection with *Streptococcus pyogenes* A (of which spiders are not vectors), that in severe cases can cause a toxic shock syndrome leading to death. The unverified bite, along with the unusual clinical presentation and the pre-existing comorbidities, makes the necrotic arachnidism unlikely and the claim of “first fatal case described in Europe” unsupported and unjustified.

Another exemplary case of unverified diagnosis is that reported by Zink et al. [27], who ascribed two necrotic skin lesions to a *Dysdera* sp*.* bite (a spider genus not considered of medical significance) solely on the finding of a dead specimen in the patient’s house more than two weeks after the onset of the symptoms and despite the detection on the ulcers of abundant *Pseudomonas aeruginosa*, of which spiders are not known carriers.

Misdiagnosing a dermonecrotic wound as a spider bite can lead to delays in appropriate care and cause adverse or even fatal outcomes. Although general wound care may be sufficient for most wounds, it is ineffective for some conditions, such as Lyme disease, necrotizing fasciitis, cutaneous neoplasms and cutaneous anthrax (a case of cutaneous anthrax, at first misdiagnosed as a brown recluse spider bite, has been reported in the US) [28], all of which need a specific treatment. Additionally, a misdiagnosis may lead to unnecessary or even harmful therapy (such as surgical excisions and administration of drugs with significant adverse effects) and may have medical-legal implications. For instance, recently, a man took legal actions because in 2017 he was misdiagnosed with a violin spider bite in a hospital in Calgary, delaying the actual diagnosis of a basal cell carcinoma by months [29].

In a 2011 study conducted in a Californian Emergency Department, patients whose major complaint was a “spider bite” were given a 12-question multiple-choice survey, in which they were asked why they believed their condition was caused by a spider bite, whether they felt a bite or sting, whether they saw a spider and what kind of spider they thought it was [30]. Interestingly, of 182 subjects, only four (2.2%) saw a spider actually biting them and 13 (7.1%) saw a spider around the time of the bite. When asked why they thought a spider was responsible for their condition, most subjects could not provide a reasonable answer, stating that some friend or relative told them it was a spider bite, that they assumed so, that they looked it up on the internet etc. When offered a choice of different spider species, more than 25% of subjects indicated instead that they had been bitten by an “other bug” or that they didn’t know if it had actually been a spider (these subjects identified their problem as a spider bite presumably for lack of better terminology). After evaluation by a physician, only seven subjects had their claim confirmed, while nine were diagnosed with bites or stings from other animals. The most notable report in this study is that as many as 152 patients (83.5%) were in the end diagnosed with skin and soft-tissue infections.

Concerningly, misinformation on spider bites also affects the medical literature. Stuber and Nentwig [31] analyzed the reliability of 134 medical reports on spider bites published in 91 journal articles and found that only 22% of them concerned verified bites. Most of these articles lack details on clinical course, therapy and healing process, therefore have no scientific value and by suggesting incorrect conclusions may even be dangerous.

## General Bite Management

The general management and treatment of any unknown spider bite should be conservative and aimed at easing the symptoms. It should include wound cleansing, elevation of the bitten extremity, application of cool compresses, tetanus prophylaxis and administration of analgesics and antihistamines (or corticosteroids). In case of a bite from any unidentified spider, the Poison Centre of Milan recommends both topical and systemic antibiotic therapy [pers. comm.]. According to Isbister and Gray [32], secondary wound infections following a spider bite are uncommon (0.9% of cases), therefore antibiotic therapy is indicated only in cases of clear signs of infection and should be based on culture and sensitivity testing [33].

## *Latrodectus tredecimguttatus*

### Introduction 

*Latrodectus tredecimguttatus* is a spider belonging to the same genus as the notorious *L. mactans* found in North America (of which it was previously considered a subspecies). It is often said to be less dangerous than its American relative because it is less likely to attack humans and encounters are uncommon, but its venom is similarly potent [34]. It is known as the European or Mediterranean black widow because it is mainly found in countries bordering the Mediterranean Sea (Spain, France, Italy, Greece), but its distribution is actually wider and ranges from the Iberic peninsula to southeastern Europe and Central Asia. It is also present in China. In Italy, where it is commonly called “malmignatta”, it lives beneath rocks or amid low scrubs on dry lands, mostly in rural areas of southern regions and Sardinia (where it is rather called “argia”). Adults can be found from June to July. Considering that this spider is not aggressive towards humans nor synanthropic (unlike its American relative), the individuals with the highest chance of being bitten are farmers (especially during the harvest), which, if elderly, are at a higher risk of complications. Similar observations were made in other countries, where most reported bites occurred in July during fieldwork or gardening [35]. Only bites of females are medically significant, since the chelicera of males are too small to pierce the skin.

Description 

**Figure 2.**
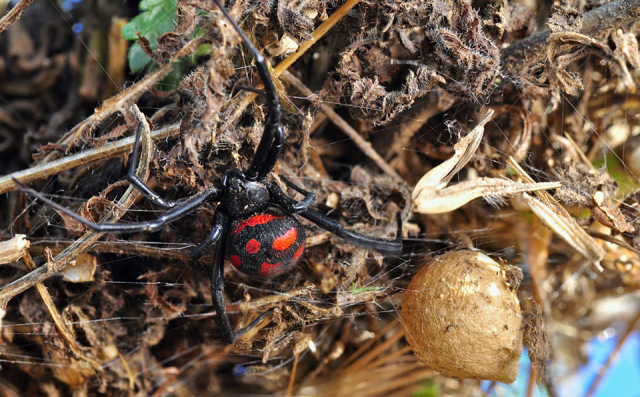
*Latrodectus tredecimguttatus* (photo courtesy of Pierluigi Rizzo).

Females can reach a body-length of up to 15 mm and are characterized by black glossy cephalothorax and legs. The abdomen is black, covered with long and short hair, marked anteriorly by a semilunar red blotch and dorsally by three longitudinal series of rounded red blotches (the series on the sides usually have three elements, while the middle one has four) (Figure 2). On the ventral surface, an hourglass-shaped figure can be present in males, while in females it is faded into just one or two bands. In younger specimens, the red blotches have a white border [36]. The specific epithet of this spider refers to the number of marks, which are 13 in total; however, this number can be lower due to the absence or fusion of some of them.

### Venom properties

The venom of *L. tredecimguttatus* is a complex mixture of proteins with different biological activities. Its main active components are the so-called latrotoxins, of which α-latrotoxin (α-LTX) is selectively toxic for vertebrates, acting both on neurons and endocrine cells. In neurons, α-LTX induces a massive release of neurotransmitters both with Ca^2+^-dependent and Ca^2+^-independent mechanisms. The toxin assembles into tetrameric complexes that bind to some plasma membrane receptors (neurexin, LPH1 and PTPσ) to form pores permeable to cations and small proteins. The binding of α-LTX to latrophilin 1 (LPH1), which is a G protein-coupled receptor, also activates a pathway that leads to the release of Ca^2+^ from intracellular stores, further promoting the exocytosis of synaptic vesicles [37]. The effects of the venom, however, are not fully explained by α-LTX alone and other components may play a critical role. Researchers of the Hunan Normal University, after collecting pure venom by electrical stimulation of living adult females, identified, apart from ten already known proteins (including latrotoxins), 33 enzymes (of which 13 were hydrolases), 23 proteins with binding function, 25 proteins with unknown function and 31 other proteins (including tryptase inhibitor, venom allergen antigen 5-like protein and fucolectin) [38].

### Clinical features

If a sufficient amount of venom is injected, the bite of *Latrodectus tredecimguttatus* can result in a clinical picture known as latrodectism, which is characterized by local and systemic symptoms. It usually does not require hospitalization, but some individuals (especially children and the elderly) may require it due to the severity of their clinical picture. The bite can be mildly to severely painful and most frequently occurs on feet, hands or limbs. Local symptoms - such as itching, tingling, hyperemia, paresthesia and decreased skin sensitivity - start to develop within 30 minutes, followed by muscle spasms and cramps, fasciculations (rare), mild fever and other systemic symptoms. The bite site typically has a “target” appearance, with a pale central area surrounded by an erythematous halo. Local or regional pain is present in 90% of cases and has an onset between 15 minutes and 1 hour after the bite; it can radiate to other parts of the body and last for several days. Abdominal (35%) and back pain (45%) can also be present. 

A peculiar feature is localized or regional diaphoresis (55% of cases). Other symptoms include excessive salivation and lacrimation, mydriasis, nausea, vomiting, headache, dizziness, asthenia, abdominal rigidity (that can mimic an acute abdomen), respiratory symptoms (such as dyspnea or tachypnea), bradycardia, hypertension, restlessness, anxiety and priapism (unusual). Patients can also exhibit the “facies latrodectismica”, characterized by flushing, a grimacing expression due to the spasm of facial muscles and periorbital edema (that can mimic an allergic reaction) [35, 39, 40, 41, 42]. Possible laboratory findings include leukocytosis, metabolic acidosis and increased levels of CPK, CRP, creatinine and troponin [42], which indicate heart involvement (*L. tredecimguttatus* is, in fact, the only species of its genus whose bite has been associated with myocardial injury) [18, 43, 44, 45]. The majority of patients with envenomation recover fully within a week. Bites resulting in death are rare [18, 19].

### Bite management

The local wound care of *Latrodectus* bites should include wound cleansing, ice packs application, oral or parenteral analgesics administration and tetanus prophylaxis. Symptomatic children, pregnant women and elderly patients with hypertension or coronary artery disease should be hospitalized for observation [33]. Until the 21^st^ century, calcium gluconate was considered a first-line treatment for latrodectism, a belief strengthened by its efficacy *in vitro* and by several flawed studies carried out in the 80s (see Ryan et al. [46] for their analysis). However, subsequent studies, such as that of Clark et al. [47], based on a larger number of cases, made its frequent use and clinical efficacy questionable. According to this study, calcium gluconate was ineffective with most moderate to severe envenomation cases, while the most effective treatment was that with IV or IM opioids and benzodiazepines (as muscle relaxants), associated with antivenom in the more severe cases. 

The administration of the antivenom shortened considerably the time needed to achieve symptomatic relief: patients treated with 1-2 vials had a complete resolution of symptoms in about 30 minutes from the end of the infusion. The intravenous route of administration was found to be the most effective in several studies (see Ryan et al. [46] for their analysis). Despite its efficacy, as of now, the antivenom therapy is indicated only in severe cases of latrodectism, due to the high risk of serum sickness and anaphylaxis (especially with equine-derived antibodies). Currently, an antivenom called Analatro® is in phase III clinical trials and should have fewer adverse reactions. According to Diaz [33], 1-2 vials of antivenom should be diluted in 100-250 mL of 0.45% NaCl solution and infused intravenously over a period of 1-2 hours. The cross-reactivity is excellent among *Latrodectus* species [46, 47, 48, 49] and it is effective in cases of severe *Steatoda* envenomation as well [50, 51]. Di Paola et al. [52] reported that the antivenom was used to treat a 60-year-old man who was bitten by a *L. tredecimguttatus*. There was no spider collection or sighting to confirm the diagnosis; however, the man showed symptoms highly suggestive of latrodectism, which resolved within an hour of intravenous infusion.

Even though calcium gluconate is considered ineffective, in several cases of *Latrodectus* envenomation (such as that reported by Calista et al. [41]), it was administered regardless, indicating a lack of awareness on current therapeutic indications for spider bites.

## *Loxosceles rufescens*

### Introduction

*Loxosceles rufescens*, known as the violin spider (see below for further explanation), is a species native to Southern Europe which, for a few decades, has shown some spreading tendencies within Europe and has been introduced to America, Australia and Asia [5]. It lives in dry and warm habitats, under stones and in rock crevices, from which it crawls out at night, but it is often found around and inside houses and buildings (behind furniture, piled boxes etc.), which offer mild temperatures that allow it to thrive all year (adult females are especially seen in spring). 

The marked synanthropy of this species and its tendency to hide in shoes, clothes and bed sheets are the main reasons its bites are frequently reported. Like most spiders, it is not aggressive, but it can bite if it is pressed against the skin (which can easily occur while dressing or sleeping). The violin spider is a close relative of the infamous *Loxosceles reclusa* (North America) and *Loxosceles laeta* (South America), accountable for many reports of severe dermonecrosis and systemic envenomation. Interestingly, specimens of *L. laeta* have been found inside the building of the Finnish Museum of Natural History in Helsinki since the 1960s [53].

### Description

**Figure 3.**
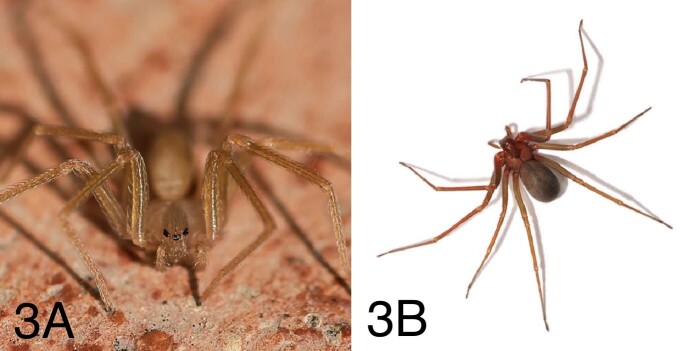
(A) *Loxosceles rufescens* (photo courtesy of Gianluigi Foggia). (B) *Loxosceles rufescens* (photo courtesy of Pierluigi Rizzo).

*Loxosceles rufescens* is an unremarkable small spider, with a body length of up to 9 mm (Figures 3A and 3B). It possesses long and thin legs, usually flattened laterally in a typical posture. The cephalothorax is light brownish (with a darker blotch on the front, shaped vaguely like a violin with its neck pointing backwards - hence the common name of the spider) whereas the abdomen is greyish or yellowish. Instead of eight eyes like most spiders, it has six, arranged in three pairs (a distinctive trait of the family Sicaridae, although not exclusive) [5, 54].

### Venom properties

The venom of *Loxosceles rufescens* contains a wide variety of proteins. The most studied components are a group of enzymes of the phospholipases-D family known as sphingomyelinases-D (SMaseD), which are present in a multitude of isoforms and catalyze the hydrolysis of the membrane phospholipid “sphingomyelin”, resulting in the formation of choline and ceramide 1-phosphate. This reaction can induce inflammation, activate the complement system and result in dermonecrosis, hemolysis, thrombocytopenia and renal failure in mammals. While SMaseD are the causative agent of dermonecrotic lesions, the pathogen *Clostridrium perfrigens* has been shown to worsen them [8]. The venom also includes astacin-like metalloproteases (loxolysin A and loxolysin B), hyaluronidases, alkaline phosphatases, serine-protease inhibitors, esterases, histamine-releasing factors, insecticidal peptides and allergen-like toxins. These components act synergistically, therefore the detailed mechanism of action of the venom is still unknown. 

The venom of *L. rufescens* is deemed far less dangerous than that of its relatives (such as *L. reclusa* and *L. laeta*), but the toxicity profiles appear similar in different species. Hence, the minor dangerousness of *L. rufescens*, if true, might be ascribable to other factors (such as shyness of the spider, limited number of case reports and dose of injected venom) [55, 56].

### Clinical features

The bite of *L. rufescens* can result in a clinical picture known as loxoscelism and 3D), of which two forms are known: cutaneous and viscero-cutaneous. The bite is usually painless, most of the time occurring while dressing or sleeping and might go unnoticed or be referred to as a pricking sensation that initially does not concern the patient. The bite site is most commonly on lower limbs, but several cases of necrosis of the eyelid have also been reported [57, 58]. 

The first symptom is a hitching or a burning sensation, occurring within a few hours, followed by the appearance of edema, erythema and a painful reddish blister, around which a cyanotic bluish area with ischemic borders appears later. The surrounding tissue is intensely erythematous. These findings give the lesion a typical blue-white-red concentric pattern. The pain gradually increases, sometimes becoming unbearable. The cyanotic area gradually turns darker and larger, sometimes acquiring a geographical appearance, and its center becomes necrotic within a few days. The erythema extends as well. The intensity of the necrosis depends on the quantity of venom injected and on the type of tissue affected (the adipose tissue suffers the most severe effects). The lesion, which usually worsens despite treatments, ulcerates in an eccentric pattern, with increasing pain and eschar formation, while peripheral inflammation signs decrease. The healing is slow and can be spontaneous or require surgical debridement, with full healing taking from 2-5 weeks to months. The process sometimes results in scarring and residual neuropathic pain [58, 59, 60, 61]. 

In less severe cases, the bite induces only an inflammatory reaction, with no necrosis, and erythema and edema disappear in 2-3 weeks [26], occasionally with the formation of an eschar that later detaches spontaneously [26, 57]. Systemic symptoms are reported in nearly half of all cases and include fever, nausea, headache, shivers, dizziness, myalgia and asthenia [26, 59]. A case of acute generalized exanthematous pustulosis has also been reported [12]. Usually there is no relevant laboratory finding in cutaneous loxoscelism. The viscero-cutaneous form is characterized by thrombocytopenia, hemolytic anemia, hemoglobinuria, myoglobinuria, acute renal failure and disseminated intravascular coagulation [33].

### Bite management

The local wound care of *Loxosceles* bites should include wound cleansing and appropriate dressing, application of ice packs, elevation of the bite extremity, administration of oral or parenteral analgesics and tetanus prophylaxis. Any patient manifesting signs of severe dermonecrosis or systemic loxoscelism should be hospitalized for observation and treatment [33]. Oral or topical antihistamines and corticosteroids are safe and effective in limiting the symptoms, but they do not prevent the evolution of the lesion nor accelerate the healing process, which is usually spontaneous [26, 33]. Intralesional injection of corticosteroids and early excision of bite lesions are contraindicated and could extend the dermonecrosis [33]. Early skin graft is also contraindicated, since it usually results in the grafted skin becoming necrotic [58]. Farace et al. [58] report of two patients who underwent immediate surgical treatment and then required prolonged hospitalization due to surgery complications, therefore they advise not to perform reconstruction at least until the 5^th^ week from the bite. 

Wound care should include debridement of necrotic tissues, culture-directed antibiotic therapy in case of secondary wound infections and delayed excision of eschars, with skin grafting as indicated [33]. Non-healing wounds may also benefit from negative pressure therapy [62, 63] or hyperbaric oxygen therapy [64], but their efficacy is not well documented and they have been used on wounds only allegedly resulting from *Loxosceles* bites. The orally administered leukocyte microtubular inhibitors, such as dapsone and colchicine, were initially recommended to halt the expansion of dermonecrosis. However, their efficacy in necrotic araneism has not been supported by randomized controlled trials and their use may pose significant toxicity risks [33]. No antivenoms are universally available, except in South America, and reports of the efficacy of systemically administered *Loxosceles* antivenoms have been mixed to date [31]. Lopes et al. [65] identified some small molecules capable of modulating the activity of sphingomyelinase-D (the most active component of the spider venom), which may have potential therapeutic roles in future treatment of loxoscelism.

## Other spiders

Although, as stated above, the sole two European species considered of medical significance are *Latrodectus tredecimguttatus* and *Loxosceles rufescens*, there are much more common synanthropic spiders that, occasionally, cause mild clinical pictures and are probably accountable for a number of bite accidents that go unreported. Some of these spiders were also considered of medical concern in the past because of presumptive cases and misidentifications, further proving how unreliable non-expert accounts can be. Additionally, some spiders, despite being capable of painfully biting humans, are only found outside of urban areas and encounters are rare.

### *Steatoda*

**Figure 4.**
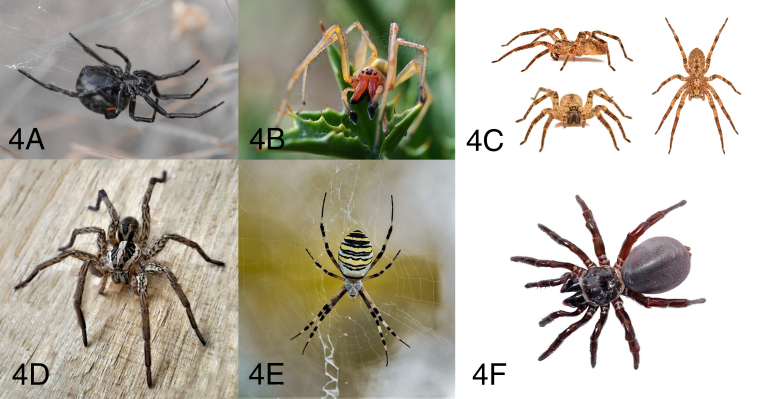
(A) *Steatoda paykulliana* (photo courtesy of Gianluigi Foggia). (B) *Cheiracanthium punctorium* (photo courtesy of Gianluigi Foggia). (C) *Zoropsis spinimana* (photo courtesy of Pierluigi Rizzo). (D) *Hogna radiata* (photo courtesy of Gianluigi Foggia). (E) *Argiope bruennichi* (photo courtesy of Gianluigi Foggia). (F) *Cteniza* sp. (photo courtesy of Pierluigi Rizzo).

Spiders of the genus *Steatoda* (commonly referred to as cupboard spiders or false black widows), such as *S. grossa* and *S. paykulliana* (Figure 4A), are cosmopolitan and can be found inside and around most buildings. They belong to the family Theridiidae, which also includes the genus *Latrodectus*, and they are easily mistaken by non-experts for black widows (hence the epithet “false black widows”). Their bite can produce moderate regional pain, erythema and swelling around the bite site, which subside within 2-3 days without complications. Systemic symptoms (including nausea, vomiting, headache, lethargy, malaise) are present in 30% of the cases; this cluster of manifestations is referred to as “steatodism”, a mild form of the latrodectism induced by their relatives. Notably, regional diaphoresis is absent in steatodism (a clinical feature that can help differentiate it from latrodectism). Since spiders of the genus *Steatoda* are synanthropic, bites usually occur indoors all year round, also during winter (this information can be useful in the differential diagnosis with *Latrodectus tredecimguttatus* bites, which usually happen outdoors during summer) [50, 66, 67].

### *Cheiracanthium*

Species of the genus *Cheiracanthium* (known as yellow sac spiders) are fast-moving spiders that sometimes can be seen indoors. They use their web to build typical sacs where they hide during daytime. These sacs are often found under leaves, in corners, clothespins and clothes (especially if left outside in rural areas or near low vegetation). Adults are usually found from August to September, when most bites have been reported [68, 69]. The time of the year must be taken into account when trying to diagnose a spider bite, since some spiders are only found during certain seasons. 

The *Cheiracanthium* bite, especially that of *C. punctorium* (Figure 4B), the largest species of this genus, can cause a sharp and burning pain, sometimes described to be as strong as that caused by a wasp or bee sting [68]. The pain usually reaches its maximum intensity after about 10 minutes and can last a few hours, with symptoms including erythema, swelling, itching, numbness and paresthesia. Systemic manifestations are rare and not life-threatening [1, 68]. From 1990 to 2011 there have been several reports of *Cheiracanthium* spp. bites in Italy [69], but some of these may have been misdiagnosed, since in one case [70] there was no identification of the spider from an expert and an atypical necrotic lesion was found. Spiders of the genus *Cheiracanthium* are frequently reported in scientific articles to be a cause of dermonecrosis in humans, but on 20 verified cases in USA and Australia and 39 reviewed international verified cases, only one showed mild signs of necrosis [71]. Hence, these spiders should not be considered of medical significance.

### Large spiders

People are often concerned about common large spiders, but this apprehension is unjustified, since their venom is not very active on humans. These spiders include *Zoropsis spinimana* (Figure 4C), *Hogna radiata* (known as the false tarantula) (Figure 4D) and *Argiope bruennichi* (known as the wasp spider for its habitus) (Figure 4E). Among these, the most likely to assume a defensive behavior and eventually bite, when disturbed, is *Zoropsis spinimana* (especially females that are pregnant or guarding their egg sac). Its bite has been described as more painful than a mosquito sting but less than a wasp sting, and causes mild symptoms such as erythema and swelling that usually resolve within a day without any specific treatment [1]. An extensive local reaction has been reported in one case [15]. 

*Tegenaria* spp. (commonly called hobo spiders) are large spiders that, similarly to *Cheiracanthium* spp., were also believed to possess a dangerous dermonecrotic venom because of presumptive and erroneous reports; however, several studies have shown this belief to be incorrect [72, 73]. Other non-medically relevant spiders that might have a painful bite due to their relatively large chelicera include non-synanthropic species such as *Lycosa* spp., *Eresus* spp., and some genera of Old World mygalomorphs known as “trapdoor spiders” - *Amblyocarenum, Atypus, Cteniza* (Figure 4F) and *Nemesia*. Concerning the genus *Lycosa*, *L. tarantula* lives in underground burrows and was unjustly held responsible in the past for the so-called “tarantism” of Southern Italy, a cultural syndrome that probably was completely unrelated to spider bites [74])

There is no official report concerning these reclusive spiders, except for some personal communications in regard to *Eresus* spp. and *Atypus* spp. In the latter case, Rezac informed Nentwig et al. [1] of bites causing pain, fever-like symptoms and increased heart rate subsiding for within two hours, headache lasting for a day and local sensitivity lasting for several days. Concerning *Atypus* spp., its bites are described to Nentwig et al. [1] by Kropf and Rezac as causing a light burning sensation with associated redness. In regard to *Lycosa* spp. and *Hogna* spp., Isbister et al. [75] state that bites of some wolf spiders cause only minor effects on humans, mostly related to mechanical trauma, which should be true for similar European species as well.

## Conclusions

Although spider bites are considered common, they are a rare occurrence, as in the majority of the works reported in the literature the actual bite by the spider has not been proved. In the Mediterranean area, the risk of serious medical consequences after a spider bite is really close to zero. However, in cases of a suspect of spider bite, a conservative treatment based on wound cleansing, elevation of the bitten extremity, application of cool compresses, tetanus prophylaxis and administration of analgesics and antihistamines should be executed. Antibiotic therapy should be indicated only in cases of clear signs of infection and based on culture and sensitivity testing. Patients that develop severe dermonecrosis or systemic symptoms should be hospitalized for observation and treatment [31]

### Abbreviations

AGEP: acute generalized exanthematous pustulosis; MRSA: methicillin-resistant *S. aureus*; SMaseD: sphingomyelinases-D.
